# Ameliorative effect of hesperidin against high dose sildenafil-induced liver and testicular oxidative stress and altered gene expression in male rats

**DOI:** 10.1186/s42826-023-00173-4

**Published:** 2023-09-21

**Authors:** Ibrahim M. Ibrahim Laila, Samar HassabAllah Kassem, Marwa Salah ElDin Mohamed Diab

**Affiliations:** 1 Molecular Drug Evaluation Department, Egyptian Drug Authority (National Organization for Drug Control and Research Formerly), Giza, Egypt; 2 Biotechnology Department, Faculty of Applied Health Sciences Technology, October 6 University, Giza, Egypt

**Keywords:** Sildenafil, Hesperidin, Oxidative stress, Antioxidants, Hepatotoxicity, Testicular toxicity, VEGF, STAR

## Abstract

**Background:**

The clinical use of sildenafil citrate (Viagra), a drug used to treat erectile dysfunction, is limited because of its many side effects on tissues. In this context, we aimed to investigate the protective effects of hesperidin, a citrus flavonoid, on hepatic and testicular damage induced by a high dose of sildenafil citrate in male rats. Rats were randomly divided into four groups. The first group was used as the control group. The second group was orally administered sildenafil citrate at a high dose of 75 mg/kg thrice a week. In the third group, hesperidin was administered orally at a dose of 50 mg/kg/day. The fourth group was administered 75 mg/kg sildenafil citrate three times a week with 50 mg/kg hesperidin daily. The experiment lasted for 28 days.

**Results:**

In the sildenafil-treated groups, blood indices were altered, liver function tests were deranged, and serum testosterone levels were reduced. In the liver and testicular tissue, sildenafil citrate treatment resulted in significant reductions in catalase and total antioxidant capacity; as well as increased malondialdehyde, reactive oxygen species, and nitrous oxide levels. In addition, sildenafil citrate treatment caused abnormal histopathological patterns in both the liver and the testes. Liver vascular endothelial growth factor and testicular steroidogenic acute regulatory protein gene expression were upregulated.

**Conclusions:**

Hesperidin attenuated the harmful effects of intensive sildenafil citrate treatment on liver and testicular functions, alleviated oxidative stress and normalized blood indices. Therefore, hesperidin could be protective against sildenafil citrate-induced oxidative damage that may develop over the long term.

## Background

Sildenafil citrate (SIL), commonly known as Viagra, is one of the most widely used and abused pharmaceutical drugs [1]. The water-soluble citrate salt sildenafil citrate was initially designed by Pfizer Company for treating angina pectoris and hypertension. Unexpectedly, this medication produced noticeable penile erection, and consequently became the standard treatment for erectile dysfunction [2, 3]. When taken prior to sexual activity, it generates consistent efficacy, good tolerance, and rapid absorption that boost its onset of action, and its plasma half-life, which lasts for 4 h, increases the duration of its action [4, 5]. SIL citrate is a specific inhibitor of phosphodiesterases 5 (PDE 5), an isozyme that hydrolyzes cyclic guanosine monophosphate (cGMP) in the corpus cavernosum [6, 7]. Its effect culminated in the accumulation of cyclic guanosine monophosphate in cells ([8, 9]. By acting on endogenous nitric oxide-cyclic guanosine monophosphate (NO-cGMP) signaling implicated in smooth muscle relaxation in the corpus cavernosum, SIL aids in raising blood flow and potentiates erectile function in humans [10–12] and murine models [13]. However, due to its widespread vasodilator effects, SIL has been associated with numerous adverse effects, including headache, congestion, and dyspepsia. The versatile biological effects of SIL have been demonstrated [14–17]. Transient hemodynamic alterations were observed after the administration of a single therapeutic dose in healthy men [14]. It has been shown that SIL can cross the blood–brain barrier [15]. Another study showed that SIL could play a role in platelet aggregation through thrombin and Von Willebrand factor (vWF) and may explain the thrombotic complications detected in some patients taking SIL [16]. Long-term administration of SIL is associated with oxidative stress, inflammation, and structural changes in the liver, heart, and testes [17].

Flavonoids are a large group of phenolic compounds widely distributed in plants. They are extensively used in traditional therapies [18]. Hesperidin (HP) as a "bioflavonoid." is broadly found in citrus fruits, especially in Citrus Aurantifolia. HP exhibits antioxidant, anti-inflammatory, anticarcinogenic, antihypertensive, and antiatherogenic properties [19]. It also exhibits potential anticancer effects [20]. Numerous studies have shown the prophylactic effect of HP against a variety of diseases, including fatty liver and cholesterolemia [21], oxidative stress in diabetic rats [22], stress-induced neurobehavioral changes [23], hepatopulmonary syndrome [24], retinal disorders, and angiogenesis in diabetic patients [25]. Moreover, it was also demonstrated that HP could have ameliorative effects on many tissues subjected to different xenobiotics, such as the liver [26], kidney [27], heart [28], brain [29], and rat hippocampus in vitro [30]. Their antioxidant properties are achieved by interacting with cell membrane lipid bilayers and protecting DNA and proteins [31]. In addition to its radical [24] scavenging properties, its antioxidant potential has been attributed to its reinforcement of cellular antioxidant defense mechanisms via the ERK/Nrf2 signaling pathway [32]. Therefore, this study aimed to investigate the prophylactic role of HP as an antioxidant and anti-inflammatory agent against SIL-induced liver and testicular oxidative damage, cytotoxicity, and gene expression alterations in male rats treated with high doses of SIL.

## Results

### Effects of HP on hematological indices

Table 1**s**hows that SIL treatment induced significant decrease (*P* < 0.05) in haemoglobin (Hb)%, red blood cell (Rbcs), hematocrit (HCT %), and prothrombin time (PT) levels of rats when compared to control group. On the other hand, HP co-treatment with SIL caused a significant ameliorative effect on blood parameters (Hb%, RBCs, and HCT%) and PT level in comparison to SIL administration alone. Hb%, RbCs, HCT%, and PT levels in HP (HP) group did not differ significantly from that of the control groups (*P* > 0.05).

**Table 1:** Effect of HP on blood Hb %, Rbcs, HCT% and PT in rats treated with SIL

	Hematology		PT (Sec)			
	Hb% (g/dl)	RBCs (106/mm^3^)	HCT (%)	Mean ± SE	Conc	INR %
Control	14.23 ± 0.23	4.90 ± 0.10	43.66 ± 0.66	12.76 ± 0.14	96	1.0
SIL	11.40 ± 0.23a	4.00 ± 0.057a	35.66 ± 0.88^a^	13.93 ± 0.06^a^	80^a^	1.2^a^
HP	14.82 ± 0.39b	5.10 ± 0.15b	45.33 ± 1.20^b^	12.60 ± 0.30^b^	96^b^	1.0^b^
SIL + HP	13.33 ± 0.61b	4.63 ± 0.22b	40.66 ± 1.85^b^	12.93 ± 0.29^b^	92^b^	1.1^b^

Values represent the mean ± SE of 6 rats. Values with the same letters are not significant at (*P* < 0.05

^a^*P* < 0.05: Significantly different from control

^b^*P* < 0.05: Significantly different from SIL-treated group

### Ameliorative effect of HP on liver and testicular functions

Serum AST, ALT and ALP levels showed significant increase (*P* < 0.05) in SIL treated group compared to control group. After 28 days, HP treatment (groups 3 and 4) significantly restored the activities of these enzymes. Additionally, a significant decrease in testosterone levels was observed in the SIL group compared to the control or HP groups. Co-administration of HP and SIL significantly rescued the concentration of testosterone to near-normal values when compared to the group fed SIL alone (*P* < 0.05) (Table. 2).

**Table 2:** Effect of HP on activities of serum AST, ALT, ALP and testosterone in SIL-treated rats

Parameters	Control	SIL	HP	SIL + HP
AST (U/ml)	30.66 ± 0.88	47.33 ± 2.18^a^	30.32 ± 1.39^b^	34.41 ± 1.38^b^
ALT (U/ml)	40.66 ± 1.76	53.18 ± 1.45^a^	40.04 ± 1.16^b^	48.21 ± 2.18^b^
ALP (U/ml)	62.66 ± 3.17	81.66 ± 2.31^a^	60.24 ± 2.15^b^	65.00 ± 1.19^b^
Testosterone (ng/ml)	3.18 ± 0.61	1.27 ± 0.38^a^	3.35 ± 0.41^b^	2.62 ± 0.28^b^

Values represent the mean ± SE of 6 rats. Values with the same letters are not significant at (*P* < 0.05)

^a^*P* < 0.05: Significantly different from control

^b^*P* < 0.05: Significantly different from SIL-treated group

### Effects of HP on oxidative stress/antioxidant enzymes activities and no in rats exposed to SIL

**Fig. 1:**
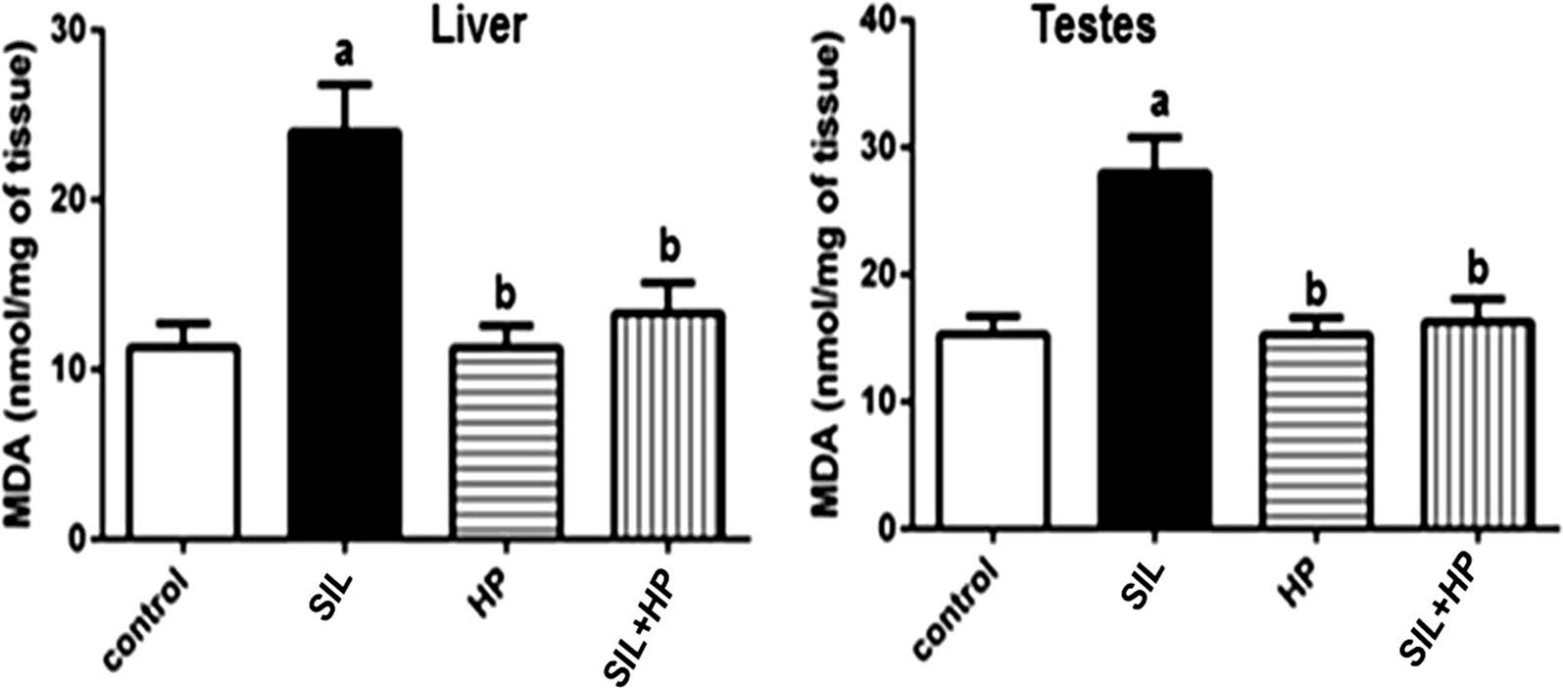
Effect of HP on MDA level in liver and testes of rat treated with SIL. Values are mean ± SE of 6 rats. Values with the same letters are not significant at (*P* < 0.05). SIL: SIL and HP: HP. a: *P* < 0.05: Significantly different from control. b: *P* < 0.05: Significantly different from SIL-treated group

**Fig. 2:**
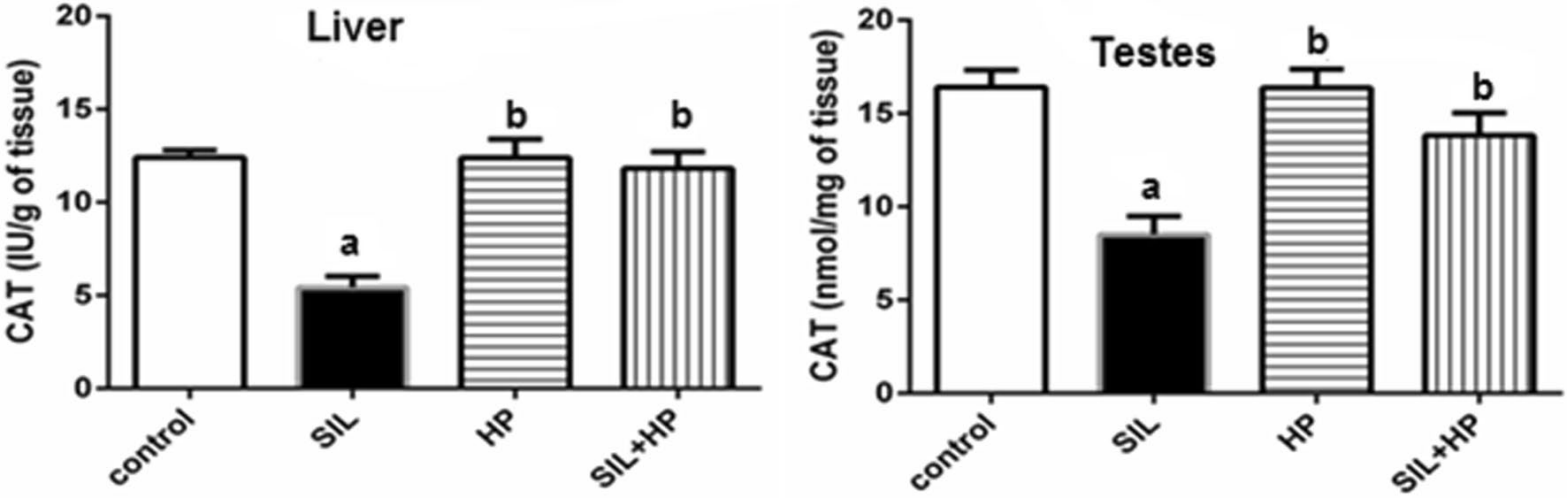
Effect of HP on CAT level in liver and testes of rat treated with SIL. Values are mean ± SE of 6 rats. Values with the same letters are not significant at (*P* < 0.05). SIL: SIL and HP: HP. a: *P* < 0.05: Significantly different from control. b: *P* < 0.05: Significantly different from SIL-treated group

**Fig. 3:**
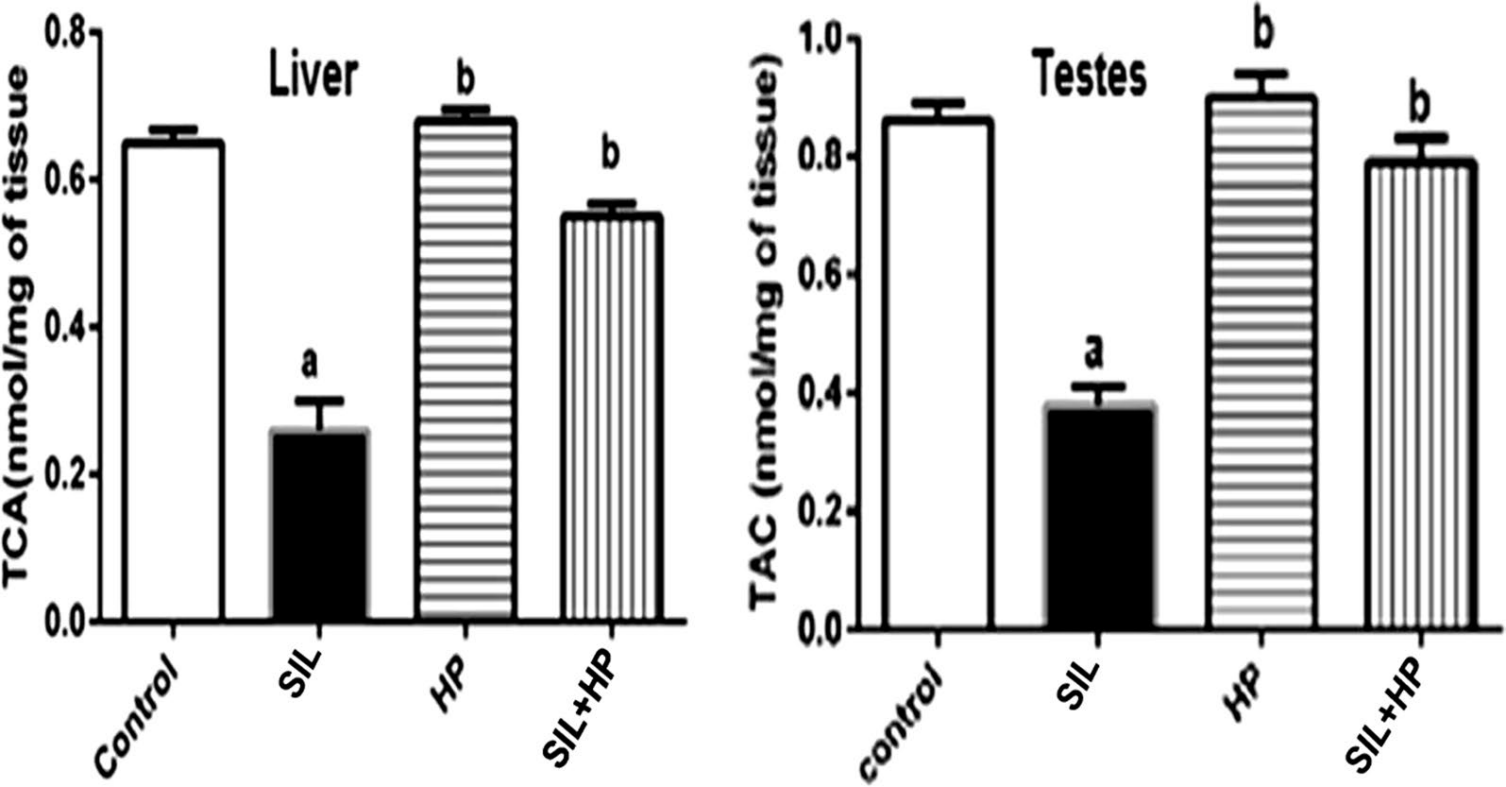
Effect of HP on total antioxidant capacity levels (TAC) in rat treated with SIL. Values are mean ± SE of 6 rats. Values with the same letters are not significant at (*P* < 0.05). SIL: SIL and HP: HP. a: *P* < 0.05: Significantly different from control. b: *P* < 0.05: Significantly different from Sild-treated group

There was a statistically significant increase in the MDA level in the SIL-treated group at the end of 28 days, compared to both the control group and the two HP groups. Notably, MDA activity was reduced significantly in the HP plus SIL-treated groups, while in the HP group, it was diminished to a level near that of the control group (*P* < 0.05, Fig. 1). The CAT levels in the rat liver and testes are shown in Fig. 2. There was no significant change in CAT activity in rats treated with HP compared to that in the control group. Treatment with SIL alone resulted in a significant decrease in Catalase (CAT) activity compared with the control (*P* < 0.05). Co-administration of SIL and HP significantly normalized its activity. A significant increase (*P* < 0.05) in the levels of reactive oxygen species (ROS) and nitric oxide (NO) in the liver and testicular tissues of rats treated with SIL was shown compared to those in HP and control groups (Table 3). Meanwhile, co-administration of HP significantly improved and normalized the activity of ROS and NO to near-control levels. HP administration maintained normal ROS and NO concentrations in the liver and testes of SIL-treated rats when compared to the control. The present study showed that SIL treatment induced a significant decrease (*P* < 0.05) in total antioxidant capacity (TAC) level of adult male rats when compared to the control group. In contrast, the SIL + HP group was significantly protected against the SIL effect on TAC compared to the SIL group alone (*P* < 0.05) (Fig. 3).

**Table 3:** Effect of HP on activities of ROS and NO levels in liver and testes of rats treated with SIL

Tr treatment g group	Liver parameters		Testes	
	ROS (μmol/g tissue)	NO (μmol/g tissue)	ROS (μmol/g tissue)	NO (μmol/g tissue)
Control	68.33 ± 5.95	200.00 ± 4.30	9.33 ± 0.69	76.33 ± 1.83
SIL	117.33 ± 4.78^a^	340.33 ± 2.86^a^	19.66 ± 0.27^a^	128.33 ± 2.45^a^
HP	65.33 ± 4.90^b^	186.43 ± 6.22^b^	10.00 ± 0.66^b^	81.33 ± 1.31^b^
SIL + HP	86.66 ± 3.76^b^	236.00 ± 3.00^b^	8.66 ± 0.89^b^	71.33 ± 1.25^b^

Values represent the mean ± SE of 6 rats. Values with the same letters are not significant at (*P* < 0.05)

^a^*P* < 0.05: Significantly different from control

^b^*P* < 0.05: Significantly different from SIL-treated group

### The protective effect HP on histopathological alterations of liver in rats fed SIL

**Fig. 4:**
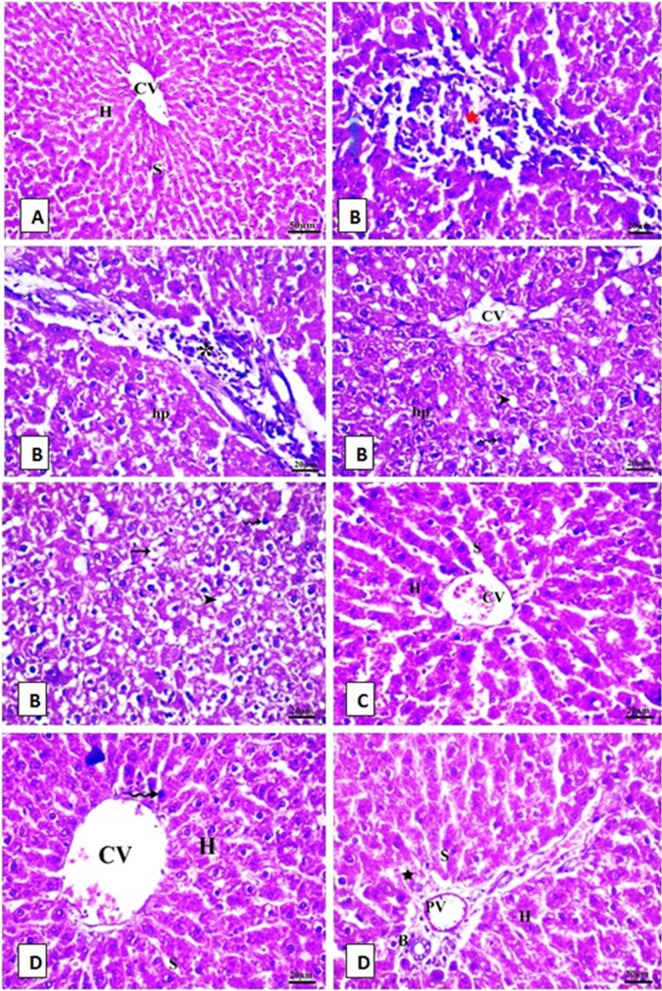
Photomicrographs of rat liver tissue stained with (H & E.X 400) for 4 groups.** A** liver of control rat showing the normal histological structure of hepatocyte (H), Sinusoid(S) and central vein (CV). **B** SIL-treated rats showing necrotic area with lymphatic infiltration (red STAR), degenerated hepatocytes (HP), pyknotic nuclei (wavy arrow), hydropic degeneration (thin arrow), fatty degeneration (Head arrow) and portal trade invaded by lymphocytes (*). **C** HP-treated group showing no histopathological alterations,** D** rats treated with SIL + HP showing bile duct, portal and slight hydropic degeneration of hepatocytes veins

Figure 4 shows a photomicrograph of sections of liver stained with H&E as follows: the control rat liver section (A) had a normal structure with no histopathological changes and also a characteristic central vein and hepatocyte arrangement. Sinusoids, the normal portal region, and similar hepatic strands spread from the edge of the lobule to the central vein were observed. In contrast, the main alterations in stained sections were recorded in the SIL group (B), as hydropic degraded cells, altered lobular shape and nuclear degradation in some places, disarrayment of typical hepatic cells, necrosis, fatty degeneration, and chromatin condensation (pyknosis) were all observed in liver sections. The hepatic central vein was enlarged and congested. Lymphocyte infiltration is observed in the portal area. Hepatocytes with cytoplasmic vacuolar balloons degenerate severely. In the HP-treated group (C), hepatocytes appeared healthy with active euchromatic nuclei. The hepatocytes and portal parts appeared normal in shape and appearance. The treatment of the SIL group with HP (D) showed evidence of improvement. The hepatic cords were correctly placed around the sinusoids, which appeared normal. Mild congestion occurs in the blood vessels. Hepatic sections showing little inflammatory cell infiltration were observed near the portal triad region. Hepatocyte degeneration was mild and there were few hepatocytes with darkly stained nuclei. The liver histopathological parameters showed abnormally high scores in the SIL group (Table 4 and Fig. 4).

**Table 4:** Score of liver histopathological examination (a semi-quantitative evaluation)

	Congestion	Lymphatic infiltration	Degeneration (pyknosis, necrosis, hydropic degeneration)	Fatty degeneration
Control	−	−	−	−
SIL	+++	++	+++	++
HP	+	−	−	−
SIL + HP	++	−	+	+

(−) indicates normal, (+) indicates mild, (++) indicates moderate, (+++) indicates high

**Fig. 5:**
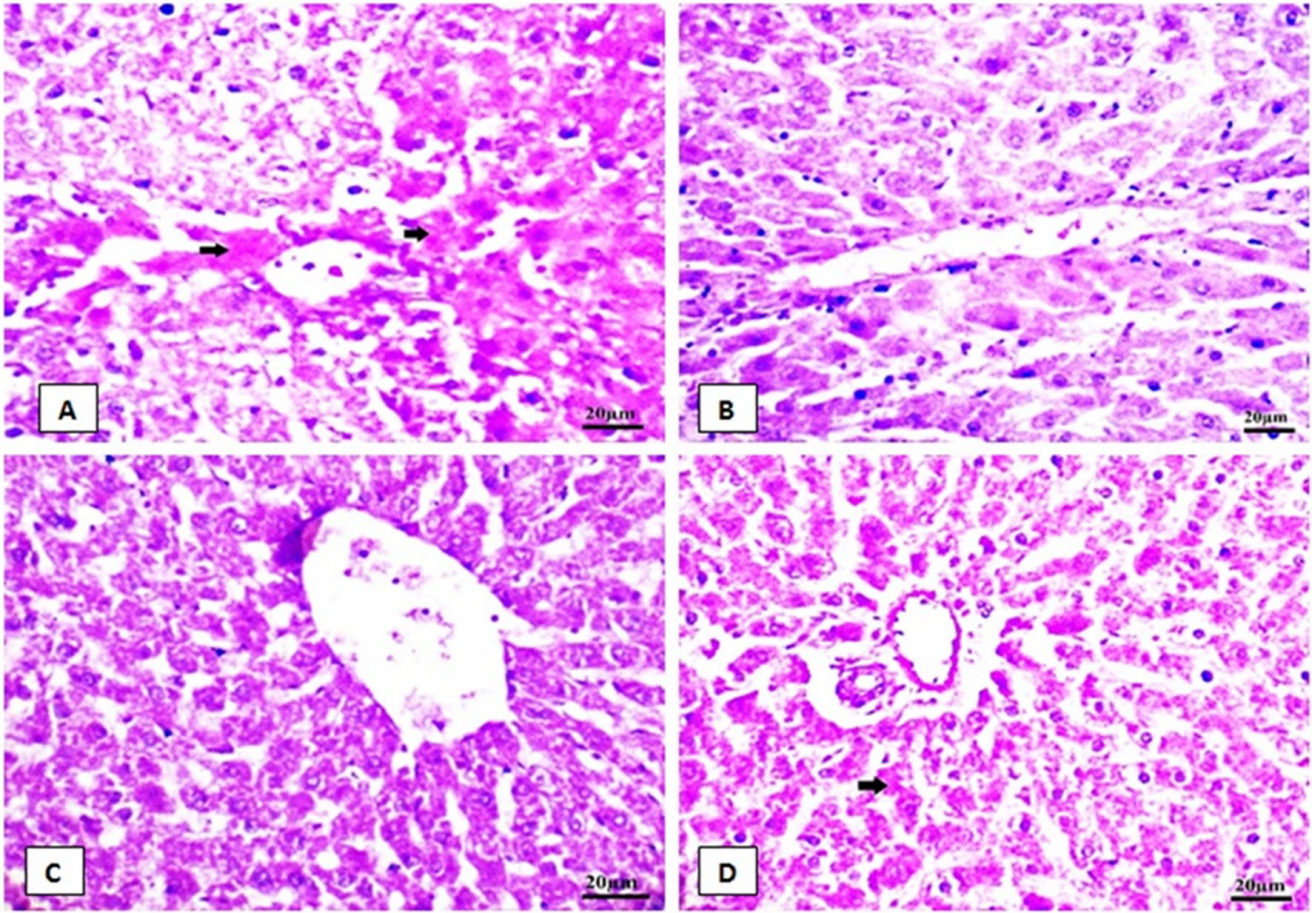
Photomicrographs of rat liver tissue stained with periodic Acid-Schiff stain (PAS) for 4 groups X 1000. **A** Control liver of rat showing high PAS positivity granules in most of the hepatocytes cytoplasm (bold arrow). **B** in SIL-treated rats, hepatocytes showed a less positive PAS reaction. **C** HP-treated group showed a high positive reaction in PAS. ** D** SIL + HP- cotreated rats showing numerous glycogen granules distributed throughout centrilobular hepatic parenchymal cells as evidenced by the presence of a high positive PAS reaction in their cytoplasm

### The protective effect of HP on histopathological alterations of testes in rats fed SIL

**Fig. 6:**
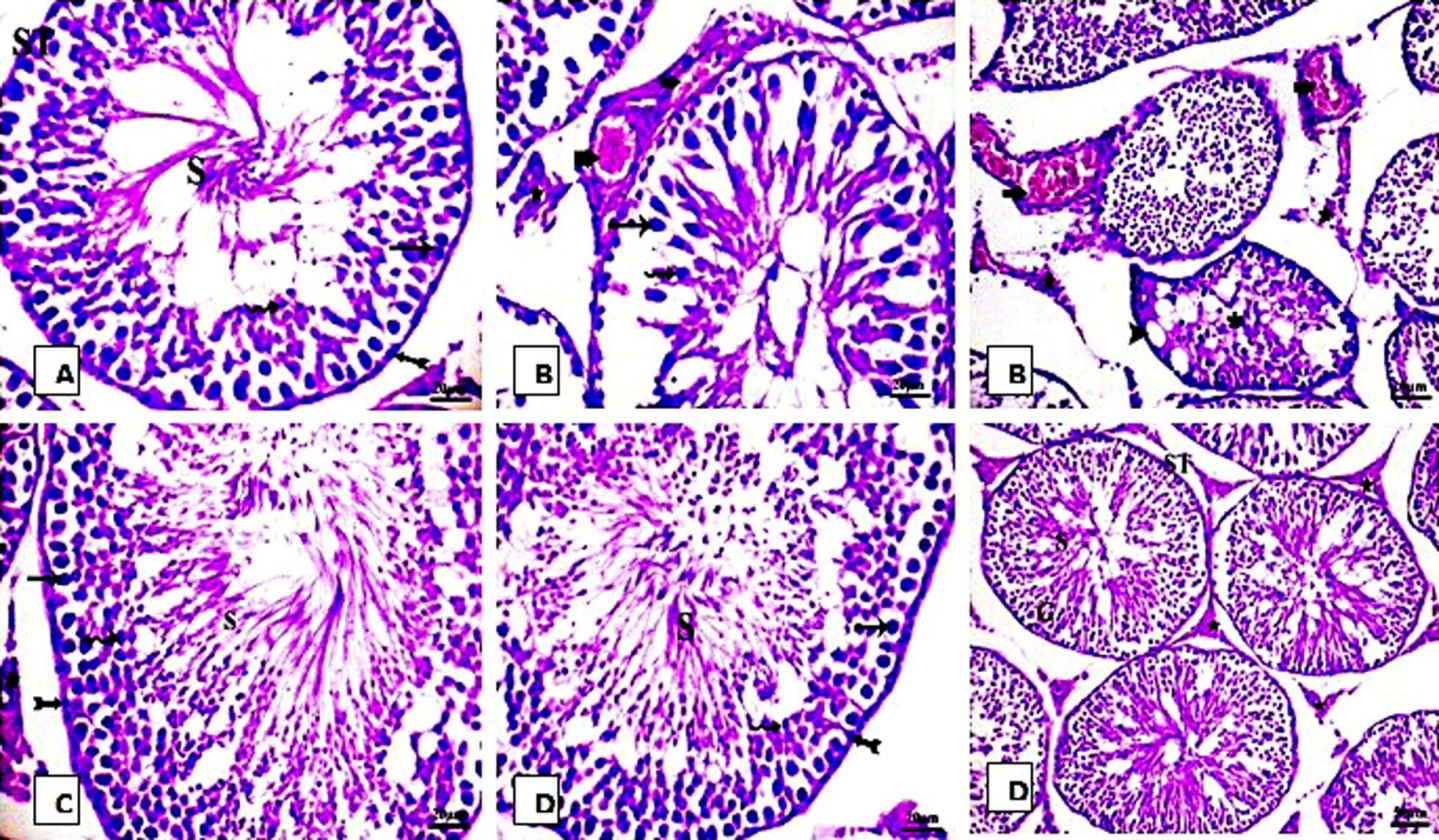
Photomicrographs of rat testis sections stained with (H & E. X 400) for 4 groups. **A** control showing the normal histological structure of seminiferous tubule (ST) with normal spermatogonia (Bifid arrow) spermatocyte, sperm(S) and spermatid (wavy arrow) **B** SIL-treated rats showing degeneration of spermatogoneal cells lining seminiferous tubules with incomplete spermatogenesis, vacuolation (head arrow), congested blood vessel (bold arrow) and interstitial hemorrhage (STAR). **C** HP-treated group showing no histopathological changes. **D** SIL + HP-co-treated rats showing normal seminiferous tubules with complete spermatogenesis and germinal cells (G)

Figure 6 showed photomicrograph of sections of testis stained with H&E as follows: The testes samples from the control and HP groups (A and C respectively) showed normal appearance of testicular tissue, which consisted of firmly organized seminiferous tubules and reflected the basic characteristics of normal structure. The spermatogenic cells in the seminiferous tubules were normally organized, with spermatogonia resting on the basement membrane, spermatocytes and spermatids, and spermatozoa within the lumen. Leydig interstitial cells were found in interstitial spaces. Testes sections from the SIL-treated group (B) showed disorganized seminiferous tubules and a significant loss in the spermatogenic cell sequence. The majority of the seminiferous tubules lacked sperm. Shedding of injured spermatogenic cells was observed within the lumen of some seminiferous tubules. The intertubular connective tissue showed a decrease in interstitial cells and widening of interstitial gaps, vacuolation, and massively congested blood vessels. In the combined group (D), there was considerable enhancement in the histological patterns. Certain seminiferous tubules contain multiple rows of spermatogenic cells at various stages. A large number of mature spermatozoa were observed in the lamina. Furthermore, some seminiferous tubules still contained spermatogenic cells that had deteriorated.

### Effects of HP on quantitative periodic acid-Schiff (PAS)-stained sections in SIL-treated hepatic rats

The cytoplasm of hepatocytes in both the control and HP groups showed a highly positive reaction in PAS stained section (bold arrow). However, in the SIL-treated group, the hepatocytes showed less positive PAS staining. In contrast, glycogen deposition was found in centrilobular hepatic parenchymal cells in the combined group, as evidenced by the presence of a highly positive PAS reaction in the cytoplasm (Fig. 5).

### Effects of HP on PAS-stained sections in SIL-treated testicular rats

**Fig. 7:**
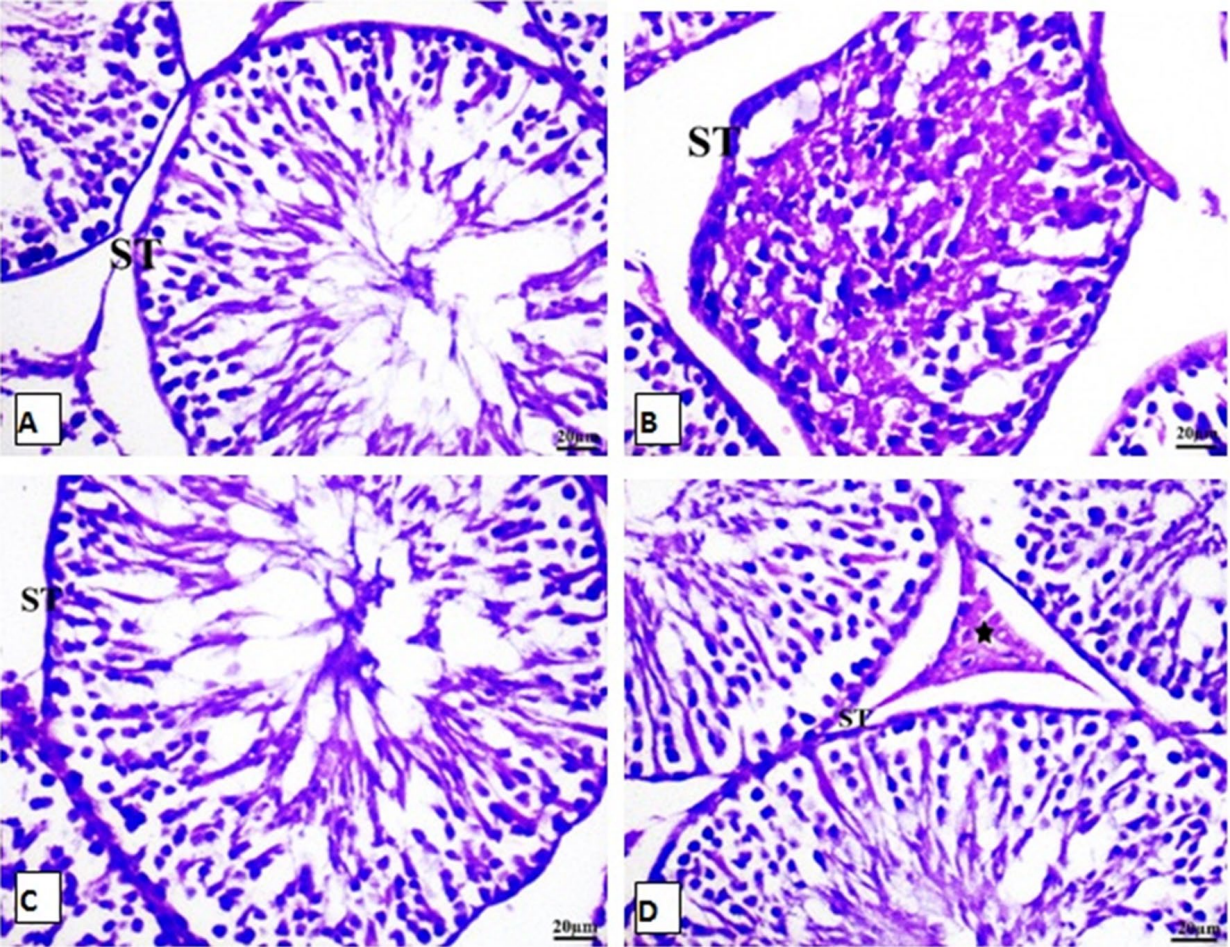
Photomicrographs of rat Testis tissue stained with PAS stain for 4 groups. **A** Control; **B** SIL-treated group **C** HP-treated group **D** SIL-treated with HP rats; no changes appeared in basement membrane thickness in the testis sections after 4 weeks of different treatments. It appeared thin and regular PAS positive membrane is seen in the control group

In the PAS-stained slides of testes, the basement membrane appeared thin and regular periodic acid Schiff (PAS)-positive membranes were observed in the control group (Fig. 7). In contrast, no changes appeared in basement membrane thickness in the SIL, HP, and combined treatment groups after 28 days.

Table 5 illustrates that oral administration of SIL for 4 weeks induced a significant (*P* < 0.05) elevation in liver VEGF mRNA expression and testicular steroidogenic acute regulatory protein (STAR) mRNA in comparison to the control group. Co-administration of HP and SIL in rats reversed gene expression alterations to near-normal levels compared to SIL-treated rats. Both gene expressions did not change significantly in HP-treated rats in the liver and testicular tissues, while the simultaneous administration of daily oral dose of HP (50 mg/kg body weight) and SIL significantly reduced their expression compared to the SIL-treated group, although it was still significantly higher than the normal level of the control group.

**Table 5:** Effect of HP on VEGF expression in liver tissues and of STAR expression in testes tissues after treatment with SIL in different groups

Parameters	QR of mRNA*	
	Liver VEGF	Testes STAR
Control	1 ± 0.01521	1.001 ± 0.0761
SIL	1.9 ± 0.01764^a^	1.82 ± 0.0954^a^
HP	0.9 ± 0.01324^b^	1 ± 0.0111^b^
SIL + HP	1.5 ± 0.01639^ab^	1.321 ± 0.0475^b^

*QR represents the relative quantification levels of the genes

^a^*P* < 0.05: Significantly different from control

^b^*P* < 0.05: Significantly different from SIL-treated group

## Discussion

In the current study, a high dose of SIL citrate was administered for 4 weeks to male rats to examine its damaging effects on the liver and testes. A high dose was selected to avoid long duration of experiments as the side effect of the drug would be prominent and to ensure the dissemination of the drug in all organs as well; thus, it may roughly approximate what is expected when the drug is administered in the long run in humans. Long-term studies are undoubtedly more realistic, but handling animals is tedious and painful, as the more we extend such an experiment, the higher the risk of animal death. We reported substantial degree of organs damage confirmed by blood indices changes, biochemical markers abnormalities and histopathological anomalies. SIL, an example of PDE5 inhibitors, was approved for the treatment of erectile dysfunction since 1996 [4]. The penile erection is dependent on the release of nitric oxide (NO) in the corpus cavernosum. SIL enhanced the effect of NO through inhibiting the degradation of cGMP [33]. Numerous studies have shown that the recreational abuses for a long time of these medications have led to several negative effects on different organs including liver, kidney, cardiac, stomach, pituitary gland and testes [34–37]. A rare case of cerebral venous sinus thrombosis (CVST) associated with long-term and high-dose SILwas reported [35]. Central retinal artery occlusion associated with SIL overdose has recently been reported [38]. Similarly, it has been shown that SIL could worsen experimentally induced abdominal aortic aneurysms [39]. Prenatal administration of SIL in animals’ models for various maternal and fetal conditions, leads to several lung and heart problems, as well as neonatal death [40, 41]. Additionally, SIL was shown to be a substantial cytotoxic agent to cancer cells [42]. All the above reports on the adverse effects of SIL citrate, although occasional, strengthened the compiling evidence of data confirming its damaging effect when administered in high and/or mild doses over an extended period. Notably, such sporadic incidences of versatile side effects may reflect the anticipated variation in individual responses.

The liver is the main site of metabolism of various substances. Alanine transaminase (ALT), aspartate transaminase (AST), and alkaline phosphate (ALP) levels reflect different functions of the liver, including anion excretion, bile production, and protein synthesis. The present study showed a significant increase in serum concentrations of liver markers AST, ALT, and ALP after 4 weeks of SIL citrate administration, which may reflect liver damage following its biotransformation by the liver. These biochemical alterations were confirmed by the liver histopathological aberrations in the SIL group. The livers treated with SIL showed an area of necrosis and vacuolization, indicative of liver damage. In agreement with the present work, it has been shown that SIL administered for 8 weeks resulted in severe hepatotoxicity, as evidenced by atrophic and degenerative changes in the liver [43]. In the current investigation, HP proved to be beneficial against SIL-induced liver toxicity. In agreement with the present study, a protective effect of HP has been demonstrated against oxidative stress produced by NaF [26], thus advocating the use of HP as an adjuvant to protect against liver damage. Supported by the wealth of literature [44], it is possible to conclude that HP can protect against liver damage induced by various oxidants. PAS commonly used to evaluate glycogen storage and function of mature hepatocytes. SIL groups showed less positive PAs stain compared to other groups, an indication of damaged and inflamed hepatocytes. These findings are consistent with earlier reports [45]. Our results showed an increase in liver VEGF mRNA expression in SIL-treated rats compared to the control and HP-treated groups. VEGF is an angiogenic peptide mediating vasculogenesis [46]. It promotes endothelial cell viability by reducing apoptosis [47]. Therefore, in our study, liver VEGF increase could reflect a defense mechanism by mediating a repair process against the damaging effects of SIL. In animal models, the use of VEGF effectively reduced ischemia reperfusion injury in a spectrum of organs, thus restoring normal conditions [48]. Numerous studies have shown that acute and chronic liver injuries activate resident stem cell niche [49]. After hepatocyte damage, the hepatic progenitor cell pool is stimulated to compensate for dead cells, thus helping to heal the hepatic injury [50]. Crosstalk between hepatocytes and endothelial cells has been demonstrated during liver damage, and this type of interaction is correlated with high VEGF expression to cope with the increased nutritional and functional demands for hepatocytes by increasing vascularization [48]. Parallel to this finding, VEGF has been shown to play a critical role in liver regeneration [50]. In the present study, HP reduced VEGF expression in the liver. Such a modulatory role points to its ameliorative effect on liver function, probably by a soothing effect on inflammatory signals. Consistent with our findings, an antiangiogenic and anti-inflammatory role of HP has been previously suggested [25]. Collectively, these results support the putative versatile protective role of HP on liver, and enhancing impact on vasculogenesis.

In the present study, serum testosterone levels were significantly lower in the SIL group than in the control and HP groups. It has been reported that testicular androgen secretion depends on both the activity and number of interstitial Leydig cells present in the testis [51]. Thus, the reduction in testosterone observed in our study could be interpreted based on Leydig cell atrophy as a result of testicular damage, and adversely affecting spermatogenesis in the SIL-treated group. Many studies have shown that SIL citrate influences spermatogenesis. In one study, reduced testosterone levels were positively associated with both sperm morphological defects [52] and motility, and semen quality [53]. It is most likely that impairment of hormonal mechanisms may affect the regulatory processes of spermatogenesis [54]. Alternatively, sperm abnormalities may be induced by oxidative stress, as the sperm plasma membrane contains a high concentration of polyunsaturated fatty acids [55]. Similarly, a significant reduction in sperm motility was observed, together with a decrease in ATP concentration and an increase in mitochondria-generated ROS, after incubation of sperm samples from healthy men with 0.03, 0.3, and 3 μM SIL citrate for 2, 3, 12, and 24 h [56]. Moreover, an increased dose of SIL caused aberrations in sperm morphology, including double-head sperm and coiled tail [37].

In parallel with the biochemical marker results, histological anomalies were observed in the testes of the SIL-treated rats. Our results are consistent with many studies that have reported a number of deleterious effects on testicular histology associated with high-dose and/or long-term administration of SIL [35, 57–60]. Similarly, deleterious histopathological changes of SIL in testis have been demonstrated, including disruption of the epithelial lining of the seminiferous tubules, destruction of the testis, impaired spermatogenesis, and the presence of inflammatory cells [61]. Contrary to our study, some studies have reported that SIL stimulates Leydig cell and testosterone secretion and improves the growth of spermatogenic cells [62–64]. Additionally, other data suggest that extensive use of sildenafil in pulmonary hypertension may not harm the prostate [65]. Another study showed its inhibitory effect on free radical generation at recommended subclinical doses [66]. Previous studies showed that low-dose SIL did not produce histological alterations in the seminiferous tubules of rats, whereas high doses resulted in degeneration, desquamation, disorganization, reduction in the germ cell population, interstitial edema, capillary congestion, and hemorrhage [57, 58]. To clarify this discrepancy, low doses of SIL may trigger a defense response to compensate for any negative effects. However, high doses may not be adequately overcome by the body's defense strategy. Thus, low to moderate doses may be beneficial for therapeutic purposes, provided that their use is limited. It is worth noting that this problem arises when prolonged sildenafil administration results in accumulating negative effects that may trigger testicular damage. In the current study, PAS stains showed no significant changes between prostate tissue sections from the control and sildenafil-treated groups. The effects of SIL may be attributed to aberrations in the expression of various cyclic guanosine monophosphate (cGMP) receptors or to the responsiveness of these receptors in the brain, which may lead to damage to testicular tissue, resulting in spermatogenesis failure [67]. Alternatively, blocking the breakdown of cyclic guanosine monophosphate (cGMP) may alter the generation of nitric oxide (NO) through negative feedback mechanism [68]. NO may have various effects on neurotransmitter activity across the brain [67], In accordance with the previous study reported the involvement of NO in erectile function, our study demonstrated a significant increase in NO in the testes of the SIL-treated group compared with both the control and HP-treated groups. The better histological patterns observed in the HP groups could reflect the enhancement effect of HP. Taken together, these findings confirm the ameliorative potential of HP against the damaging effects of SIL on testes. Furthermore, HP, as a strong antioxidant belonging to flavonoid family, could assist the testes further by improving erectile function. Groups treated with HP alone demonstrated slight but significant increase in serum testosterone and testicular NO levels compared to control. This suggests that HP may be advantageous for healthy individuals and may prevent erectile dysfunction in the future. A previous study confirmed the association between flavonoid intake and a reduced incidence of erectile dysfunction [69]. Consequently, HP turn out to have a wide range of health benefits, including both preventive and enhancing effects. Thus, the likelihood that HP could further enhances erectile function warrants additional research. Regarding STAR gene expression in the testes following treatment with SIL, a significant increase was observed compared to the control and HP-treated groups. This alteration may reflect a disturbance in sex hormone regulation during the later stages of spermatogenesis. STAR transcripts increased significantly, corresponding to the increases of serum sex-steroid levels. Alternatively, one study demonstrated that STAR abundance at least partially influences the level of steroid production during spermatogenesis in rainbow trout [70]; while other one showed that it plays a significant role in controlling testosterone production [71]. A previous mammalian study has demonstrated the induction of the STAR protein by LH treatment in rat Leydig cells [72]. Thus, testicular function decline confirmed by our histological results in SIL group could lead to reduced serum testosterone (1.27 ± 0.38a) which, subsequently triggers an increase in LH. Thus, higher STAR expression induced by the increase in LH rise is met by the lack of adequate hormonal response from the damaged testes. This might ultimately induce aberrant STAR expression in SIL-treated group. HP-treated group showed a modulatory effect on STAR expression (1 ± 0.0111). In accordance to our work, HP was shown to prevent inflammation, oxidative stress and apoptosis in testicular tissue, while exhibited a protective effect on FSH, LH, testosterone levels and modulated steroidogenic enzyme levels in the testes of rats treated with BPA; however, in contrast to the present study, STAR expression showed pronounced downregulation [73]. Oxidative stress and inflammation was demonstrated to reduce the expression of this protein [74]. In recent study, HP administration successfully reduced testicular damage caused by nickel oxide nanoparticles and modulated the expression of steroidogenesis-related genes, such as STAR [75]. Given the multitude of factors involved in testicular function, the dynamic state of modulating STAR expression reflects the complexity of real-time conditions in the testes. Hence, under our experimental conditions, the deficit of testosterone may enhance the upregulation of the STAR gene, whereas in other conditions, the state of the regulatory protein could be reversed.

Our results showed significant changes in the antioxidant status in the SIL-treated groups compared to the control and HP groups, as well as in the combined treatment groups. A reduction in antioxidant enzymes and an augmentation of lipid peroxidation were obvious in the SIL-treated groups, whereas HP administration assisted in restoring normal antioxidant enzymes and MDA levels. SIL citrate significantly increases MDA levels in the testes [66]. Moreover, in a parallel study [76], lipid peroxidation in the testes significantly increased after SIL administration, whereas all tested agents, including rutin, HP, or their combination, caused a marked decrease in this parameter. Thus, the ameliorative effects of HP on liver and testis damage induced by SIL could be mediated by restoring normal antioxidant status. In concordance with our study, HP protected against tissue damage caused by various chemical factors [76–80]. HP upregulates testicular gonadotropin receptors, steroidogenesis markers (steroidogenic acute regulatory protein), and VEGF. Additionally, it prevents testicular injury, suppresses lipid peroxidation and nitric oxide, and increases antioxidants in SIL-induced liver and testis damage [75, 81].

The rapid clearance of SIL citrate from plasma 4 h after administration may reflect a prompt diffusion rate in organs. Based on pharmacokinetic studies [81], it was suggested a SIL distribution in tissues and possible tissue binding were suggested. To our knowledge, the metabolic rates of SIL citrate in organs other than the liver microsomes, shown to be the predominant route of its disposal as described previously [82], have not been fully elucidated. However, other tissues may possess less efficient xenobiotic degradation machinery that would be amenable to residual accumulation of the drug in the long term. Taking into consideration the side effects of SIL encountered in various organs, as shown in our study and previous studies [34–42], we hypothesize that residual amounts of SIL persisting in different tissues such as arteries, brain, eyes, and testes, as a result of continuous administration, may culminate in tissue damage, possibly by perturbing PDE5 expression and cGMP metabolism. It is well-known that PDE 5 is ubiquitous. Its three isoforms, PDE5A1, PDE5A2, and PDE5A3, are inhibited by SIL [83]. This wide effect may trigger the dysregulation of PDE5-cGMP signaling in various tissues. To clarify this assumption, we can state that a consistent rise in cGMP following long-term SIL administration may temporarily upregulate PDE 5 expression as a feedback mechanism, necessitating an escalating dose of SIL to effectively suppress this increasing flux of PDE 5. In support to this claim, intracellular cGMP has been shown to increase PDE5 expression [84]. Thus, a persistent stimulation of PDE5 expression by prolonged SIL intake may ultimately result in the downregulation of this gene leading occasionally to an exacerbation condition. Deregulated cGMP metabolism has recently been demonstrated [39]. In support of our hypothesis, a tachyphylaxis effect with SIL has been reported [85], leading to increased consumption to overcome the reduction in drug efficacy. Among juvenile patients, a randomized double-blind study found a strong correlation between high-dose SIL monotherapy and mortality [86]. In addition, priapism as a complication of oral SIL citrate abuse has occasionally been reported [87, 88]. Taken together, the putative residual accumulation of SIL citrate may aggravate the various problems encountered sporadically after long-term SIL citrate use, thereby warranting serious consideration.

We did not assess the two studied genes, VEGF and Star, at the protein level, assuming this would not strengthen the validity of our study as it is beyond our research question. We aimed to assess whether the drug affects their genes expression; therefore, further studies may explore the effects of the drug at the protein level. However, this missed assessment of gene functionality could be a limitation of the present study.

## Conclusions

SIL showed impairment of liver and testicular functions and hence, limitation of unnecessary use becomes an urgent demand for good health status. HP as antioxidant, could repair efficiently the SIL-induced toxicity in testes and liver. We place great emphasis on the scope of SIL as therapeutic agent and therein, we urgently recommend a restricted use. As SIL is now over the counter in many countries, reevaluating the guideline of doses uses in different patient’s categories become urgent. For sufficient clearance of medication from tissues, dose administration at spaced-apart intervals is recommended. As high percentage of young male become abused to SIL, addressing safety concerns regarding its long-term uses on their future offspring become urgent. Additional studies are required to expand our understanding of the molecular mechanism of SIL that triggers different medical conditions. We recommend the intake of HP as a prophylactic agent, especially in vulnerable categories requiring SIL. In this context, standardization of the HP dose warrants further investigation. Clinical studies must be conducted to examine HP bioavailability, appropriate doses, and efficacy. Finally, raising cautious awareness of the possible side effects of SIL recreational use in the long run is highly demanded both nationally and internationally.

## Methods

### Materials

All the chemicals used in the experiments were purchased from Sigma Chemical Co. (Munich, Germany) except for SIL, which was obtained from Pfizer (Istanbul, Turkey) and was dissolved in distilled water for oral administration. The study was performed in the department of Molecular Drug Evaluation at the National Drug authority, Giza, Egypt.

### Animals

For the experiment, 24 mature male albino rats weighing 160–180 g were obtained from the animal house of the National Drug Authority. The animals were housed in cages under standard laboratory conditions and maintained at 22–27 °C. They were allowed ad libitum access to water and feed, exposed to a 12/12 h light/dark cycle, and allowed to acclimatize to the laboratory conditions for 1 week before the start of the experiment.

### Experimental design

The rats were divided into four groups of six rats each. Each group was housed in separate polypropylene cages (450 mm × 270 mm × 150 mm) with sawdust. The sawdust substratum was changed every alternate day. All animal procedures were performed in accordance with the recommendations of the Canadian Committee for Care and approved by the Research Ethics committee for experimental and clinical studies of Applied Health Sciences Technology, October 6 University (Approval Number: 20220515). All groups were treated as follows: Group 1 (Control), rats received 1 ml of normal saline solution; group 2 (SIL), rats received 75 mg SIL orally thrice a week; group 3(HP), rats orally received 50 mg/kg HP daily; group 4 (SIL + HP), rats were orally administered 75 mg/kg SIL thrice a week, together with 50 mg/kg HP daily. After 28 days of treatment, under anesthesia with diethyl ether, animals were euthanized. Blood samples were immediately collected from the orbital sinus in two centrifuge tubes for each animal, one tube containing ethylenediaminetetraacetic acid (EDTA) for measuring blood indices, and the second tube was left at room temperature for 40 min. From the second tube, sera were separated by centrifugation at 3000 rpm for 10 min and kept frozen at − 20 °C for biochemical assays. The liver and testes of each animal were dissected into two parts. One part was fixed in 10% formalin for 24 h for histopathological examination using hematoxylin and eosin staining. The second part of the liver and testes was removed, washed with 0.9% NaCl solution, and stored at − 80 °C until use. One gram of each sample was homogenized in 10 ml saline solution using a Teflon homogenizer (Glas-Col, Terre Haute, USA) for different biochemical assays in liver and testis tissues.

The hemoglobin (Hb%) concentration, red blood cell (RBC) count, and hematocrit (HCT) were determined using a semiautomatic hematological analyzer (SWELAB IEO Model, UK). The auto counter used 20 μl of blood in 16 ml of a commercially prepared diluent.

### Blood biochemical analysis

Aspartate aminotransferase (AST) and alanine aminotransferase (ALT) activities in serum were measured using standard protocols [89]. Alkaline phosphatase (ALP) activity was determined calorimetrically in serum [90]. Testosterone levels were determined using enzyme-linked immunosorbent assay (ELISA) kits (BIOS, Microwell Diagnostic System, USA).

### Oxidative stress analysis

Total antioxidant capacity was determined calorimetrically in homogenized tissues using a kit provided by Biodiagnostics, Cairo, Egypt [91]. MDA content was determined using the thiobarbituric acid (TBA) test [92]. Tissue CAT activity was measured as previously described [93]. A modified version of a previously described assay for the intracellular conversion of nitro blue tetrazolium (NBT) to form azan by superoxide anions was used to measure the generation of reactive oxygen species ROS [94]. NO levels were estimated using the Griess reaction [95]. Testosterone levels were determined using enzyme-linked immunosorbent assay (ELISA) kits (BIOS Microwell Diagnostic System, USA).

### Histopathological analysis

For histopathological examination, liver and testis specimens were fixed in 10% neutral buffered formalin for 24 h and examined under a light microscope (Zeiss model, Oxford instruments). Tissue specimens were embedded in paraffin wax using a conventional method and subsequently stained with Harris hematoxylin and eosin stain as well as periodic acid-Schiff stains (PAS) for histopathological studies. The slides were examined under a light microscope. Periodic acid-Schiff staining analysis of liver sections from both control and treated rats. The accumulated glycogen appears purple (a). Scale bars = 100 μm. PAS stain was scored based on the intensity of the positively stained cells, which ranged from 0 (0–15%), 1 (16–25%), 2 (26–50%), or 3 (76–100%). Quantitative periodic acid-Schiff (PAS) staining analysis of treated rat livers depending on the area fraction [45].

In addition, a semiquantitative assessment of liver histopathology was performed using the following scoring system: zero: normal; one: mild; two: moderate; three: severe. The average score for each group was calculated to determine the total score.

### Quantitative real-time polymerase chain reaction analyses (qRT-PCR)

The effect of HP on the expression of hepatic vascular endothelial growth factor (VEGF) and testicular steroidogenic acute regulatory protein (STAR) was studied using quantitative RT-PCR. Total RNA was isolated from liver and testis homogenates using RNase purification reagent (Qiagen, USA), according to the manufacturer’s instructions, and quantified at 260 nm. RNA concentration was measured using a spectrophotometer (Dual-Wavelength Beckman, Spectrophotometer, USA). Five micrograms of RNA were reverse transcribed to produce cDNA using oligonucleotide (dT) 18 primer (final concentration, 0.2 mM) (Table 6), and then denatured at 70 °C for 2 min. Denatured RNA and reverse transcription mixture buffer (50 mM Tris HCl (pH 8.3), 50 mM KCl, 0.5 mM deoxyribonucleotide triphosphate (dNTP), 1 U/mL RNase inhibitor, 3 mM MgCl2, and 200 units of Moloney Murine Leukemia Virus reverse transcriptase) were placed on ice. The reaction tubes were then incubated at 42 °C for 1 h. This was followed by heating to 92 °C to stop the reaction.

**Table 6:** Primer sequences used in this study were designed as follows

Gene	Primer Sequence
VEGF	sense 5′-TAGGGTTTTTTTCAGTATTCT-3′ antisense 5′-TTTTCTTGTTTTGTTTTTACAT-3′
STAR	Sense: 5′-TTGGGCATACTCAACAACCA-3′ Antisense:—5′ATGACACCGCTTTGCTCAG-3′
β-actin (as internal control)	Sense: 5′-GAGAGGGAAATCGTGCGTAC-3′ antisense: 5′-CATCTGCTGGAAGGTGGACA-3′

Five milliliters of first-strand cDNA was used in a total volume of 25 μL for real-time quantitative PCR, containing 12.5 μL 2 × SYBR Green PCR Master Mix (Applied Biosystems, Foster City, CA, USA), and 200 ng of each primer, as shown in Table 6. PCR reactions, consisting of 95 °C for 10 min (1 cycle), 94 °C for 15 s, and 60 °C for 1 min (40 cycles), were performed using a real-time PCR system (Rotorgene RT-PCR system, Rotor-Gene 6000 Series Software 1.7 (Quiagen). Data were analyzed using ABI Prism 7500 sequence detection system software and quantified using v1 7 Sequence Detection Software from PE Biosystems (Foster City, CA). The relative quantification levels (QR) of the 2 studied genes were calculated using the comparative threshold cycle technique. All values were normalized using the β-actin gene as an internal reference gene [96]**.**

### Statistical analysis

Data are presented as means ± SEM. Groups of data were compared using one-way analysis of variance (ANOVA) followed by the post hoc Tukey-HSD test for multiple comparisons of the means and the statistical significance was set at *P* < 0.05.

## Data Availability

All data are given in the current manuscript.

## Abbreviations

SIL: Sildenafil citrate
HP: Hesperidin
AST: Aspartate aminotransferase
ALT: Alanine aminotransferase
ALP: Alkaline phosphatase
VEGF: Vascular endothelial growth factor
STAR: Steroidogenic acute regulatory protein
TOC: Total antioxidant capacity
ROS: Reactive oxygen species
MDA: Malondialdehyde
CAT: Catalase
PT: Prothrombin time
vWF: Von Willebrand factor
